# Ecosystem Evapotranspiration as a Response to Climate and Vegetation Coverage Changes in Northwest Yunnan, China

**DOI:** 10.1371/journal.pone.0134795

**Published:** 2015-08-03

**Authors:** Hao Yang, Peng Luo, Jun Wang, Chengxiang Mou, Li Mo, Zhiyuan Wang, Yao Fu, Honghui Lin, Yongping Yang, Laxmi Dutt Bhatta

**Affiliations:** 1 Key Laboratory of Mountain Ecological Restoration and Bioresource Utilization of Chinese Academy of Sciences, Chengdu Institute of Biology, Chinese Academy of Sciences, Chengdu, China; 2 College of Life Sciences, Sichuan University, Chengdu, China; 3 Ecological Restoration and Biodiversity Conservation Key Laboratory of Sichuan Province, Chengdu Institute of Biology, Chinese Academy of Sciences, Chengdu, China; 4 University of Chinese Academy of Sciences, Beijing, China; 5 Yunnan Academy of Agricultural Sciences, Kunming, China; 6 Kunming Institute of Botany, Chinese Academy of Sciences, Kunming, China; 7 International Centre for Integrated Mountain Development, Kathmandu, Nepal; Peking University, CHINA

## Abstract

Climate and human-driven changes play an important role in regional droughts. Northwest Yunnan Province is a key region for biodiversity conservation in China, and it has experienced severe droughts since the beginning of this century; however, the extent of the contributions from climate and human-driven changes remains unclear. We calculated the ecosystem evapotranspiration (ET) and water yield (WY) of northwest Yunnan Province, China from 2001 to 2013 using meteorological and remote sensing observation data and a Surface Energy Balance System (SEBS) model. Multivariate regression analyses were used to differentiate the contribution of climate and vegetation coverage to ET. The results showed that the annual average vegetation coverage significantly increased over time with a mean of 0.69 in spite of the precipitation fluctuation. Afforestation/reforestation and other management efforts attributed to vegetation coverage increase in NW Yunnan. Both ET and WY considerably fluctuated with the climate factors, which ranged from 623.29 mm to 893.8 mm and –51.88 mm to 384.40 mm over the time period. Spatially, ET in the southeast of NW Yunnan (mainly in Lijiang) increased significantly, which was in line with the spatial trend of vegetation coverage. Multivariate linear regression analysis indicated that climatic factors accounted for 85.18% of the ET variation, while vegetation coverage explained 14.82%. On the other hand, precipitation accounted for 67.5% of the WY. We conclude that the continuous droughts in northwest Yunnan were primarily climatically driven; however, man-made land cover and vegetation changes also increased the vulnerability of local populations to drought. Because of the high proportion of the water yield consumed for subsistence and poor infrastructure for water management, local populations have been highly vulnerable to climate drought conditions. We suggest that conservation of native vegetation and development of water-conserving agricultural practices should be implemented as adaptive strategies to mitigate climate change.

## Introduction

Climate change had influences on biodiversity and socio-economic systems primarily through increasing temperatures and climate-related disasters [1, 2]. Improving the capacity of the environment to adapt to continuous climate change is vital for the sustainable development of human society and economic systems [3, 4]. Therefore, it is imperative to understand vulnerability of systems and employ corresponding adaptive strategies for biodiversity conservation and adaptive management. Yunnan Province, which is located in Southwest China, is a key area for biodiversity because its vast mountains host more than 18, 000 high plant species and 1, 836 vertebrate species, which account for 51.6% of the total plants and 54.8% of the total vertebrate species in China [5], respectively. Since the last decade, this area has experienced continuous and intensifying drought conditions, especially in spring, which have led to great social and economic losses [6–8]. These continuous droughts are of significant concern globally and nationally because they are a typical example of the influence of climate change on the sustainability of ecological and social systems in a biodiversity hotspot. Understanding the reasons for increased drought conditions is important for developing adaptive strategies for both conservation and development.

In general, drought can be attributed to climate and anthropogenic activities. Global aridity, especially in Africa, Southern Europe, East and South Asia, and Eastern Australia since the late 1970s has been attributed to natural variations in the El Nino-Southern Oscillation (ENSO), tropical Atlantic sea surface temperatures, Asian monsoons, and global warming [9]. Human activities have also triggered or aggravated drought by changing aerosols concentrations [10], altering land use/land cover [11–14], and increasing water demand along with social development [15]. A tendency of climate warming over the past 50 years was observed in Yunnan [16, 17], and the moisture or precipitation deficiency in combination with increasing temperatures accounted for drought conditions [18]. However, a number of studies have found that deficiencies during droughts were within the range of variations observed during historical climatic drought events [19, 20]. Therefore, the continuous droughts occurring in Yunnan could not be fully explained by climate reasons alone. Although the potential effects of human activities on droughts have been widely discussed [21–23], empirical studies have not been conducted to determine the extent of climate-change or human-activity contributions to the drought.

Drought causes shortages of available water for the ecological and social systems [24] and occurs when the ability of the ecosystem to provision water is lowered, which aggravates drought damage. The water provision function of an ecosystem can be measured by its water yield (WY), which is the sum of runoff and soil undercurrent after plant transpiration, surface evaporation, and vegetation/soil retention [25]. In addition to precipitation, evapotranspiration (ET) is the largest factor influencing WY in water balance equations, which is greatly altered by vegetation dynamics and characteristics [26]. Higher ET results in less water provision to the social system, making it more vulnerable to climatic drought. In recent decades, Yunnan underwent rapid economic development and the most dramatic land-use and land-cover changes in its history [14, 27–29]. The natural vegetation of the area has been significantly modified by increased human utilization, and most of its barren hilly lands have been re-vegetated in massive afforestation and reforestation efforts [14, 30–32]. Therefore, determining the relationship between the dynamics of ET and vegetation changes is crucial to understanding the causes of droughts and determining the increasing vulnerability of the social system to climatic drought in this hotspot area.

We hypothesized that the vegetation coverage changes induced by afforestation/reforestation and other vegetation management activities over the past 13 years have increased the water consumption (ET) and lowered the ecosystem’s water provision capability, which may eventually reduce the social system’s resilience to manage water shortage caused by climatic drought. Northwest (NW) Yunnan is an internationally recognized key area of biodiversity conservation in China [33], and it was selected as the study area to test this hypothesis. We utilized a surface energy balance system (SEBS) model to calculate the regional ET according to relevant meteorological data and remote sensing data from 2001 to 2013. We also analyzed the correlations among ET, climate, and vegetation coverage to 1) measure the ecohydrological effects of climate and vegetation coverage changes and 2) analyze and discuss the impact of climate and vegetation coverage changes on droughts in NW Yunnan. The results of this study may be used as a guide in the planning of adaptive strategies for sustainable development in this globally important area of biodiversity conservation.

## Study Area

As a part of the Hengduan Mountains, one of the 17 critical regions for biodiversity conservation and the world’s most diverse temperate mountain forests [34, 35], NW Yunnan has experienced significant vegetation changes because of rapid economic development and massive afforestation/reforestation programs (i.e., National Forest Protection Plan) [14, 30, 36]. NW Yunnan is located in the east of the Himalayas (24°38′ N–29°15′ N, 98°05′ E–101°16′ E) at elevations varying from 648 m to 6,740 m. This area is in the upstream area of four major rivers (Yangtze, Mekong, Salween, and Irrawaddy) in Asia and possesses unique ecological functions [37], especially as the water source area to over 100 million people downstream. Diverse and complicated topography in this area create a variety of microclimates [38]. In general, monsoons bring moisture in summer, with most of the precipitation falling between June and September. The mean annual temperature in this area is 12.3°C and the mean annual rainfall is 1,013 mm.

**Fig 1:**
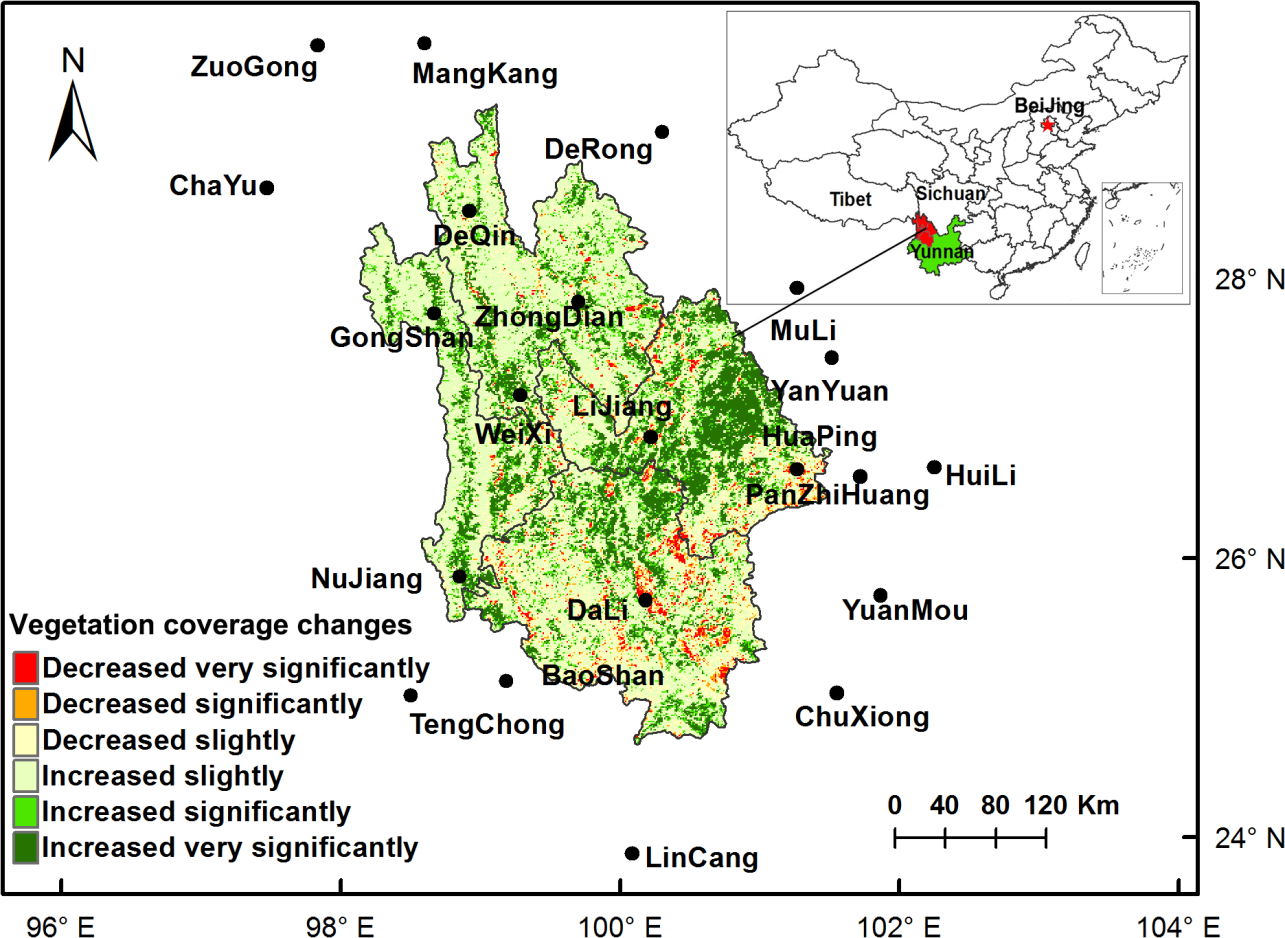
Locations of the 21 meteorological stations and vegetation coverage changes over past 13 years. The trend of vegetation coverage changes from 2001 to 2013 is estimated by linear trend analysis. F-test is employed to test significance of the trends. The increase or decrease trends of vegetation coverage are categorized into three levels: very significantly (P < 0.01), significantly (P < 0.05), and slightly (P > 0.05).

The land area of NW Yunnan accounts for only 0.4% of the total area in China; however, it hosts 20% of the high plant species and approximately one quarter to one-third of the total animals in China [39]. Evergreen broadleaf forest and mixed forest are the major forest types, alpine grassland is also common [37]. Traditional mountain agriculture is still the major livelihood for its rural population. NW Yunnan covers four administrative prefectures: Nujiang, Diqing, Dali, and Lijiang (Fig 1). Dali and Lijiang are relatively more densely populated and developed, with population densities of 118.6 and 59.5 people per km^2^, respectively. Compared with Dali and Lijiang, Nujiang and Diqing are less populated, with densities of 36.6 and 17.0 people per km^2^, respectively [40]. Continuous droughts over the last decade in NW Yunnan, especially in Dali and Lijiang, have reduced agricultural production and triggered severe domestic water shortages (Climate Communique of Dali Prefecture: http://dl.xxgk.yn.gov.cn/z_m_003/Info_More.aspx?Classid=126584).

## Data Source

Moderate Resolution Imaging Spectroradiometer (MODIS) L1B time-series data (MOD021KM, MOD03) from 2001 to 2013 were selected for the estimates of ET and WY because Terra MODIS began to send data in April 2000. All of the MODIS products were downloaded freely from the NASA website (http://ladsweb.nascom.nasa.gov/browse_images). We obtained one to three images every month for the requirement of cloud-free images or quasi-cloud-free images, which met our needs. For fraction of vegetation coverage estimation, MODIS level 3 monthly vegetation indices (MOD13A3) were download from the same website. Digital elevation model (DEM) data were obtained from the Shuttle Radar Topographic Mission (SRTM) project (http://srtm.csi.cgiar.org/SELECTION/listImages.asp) with 90 m spatial resolution, which were prepared to calculate the ET of the heterogeneous underlying surface (NW Yunnan) in SEBS.

Meteorological data were downloaded from the China Metrological Data Sharing Service System (http://cdc.nmic.cn/home.do), and data from 21 weather stations in the vicinity of the research area were obtained for 2001 and 2013 (Fig 1).

## Methods

### ET and WY calculation

WY can be calculated as the difference between precipitation and ET [41–43]. The widely used remote sensing model SEBS was chosen to calculate the ET of heterogeneous land surfaces according to satellite data and meteorological information at proper scales [44, 45]. The model is based on traditional energy-balance equations, and it successfully used in different studies worldwide [46–49]. Su et al. proposed the SEBS model [50]. The surface energy balance is commonly written as follows:

$$\lambda E=R_{n}-G_{0}-H$$ (1)

where *λE* is the turbulent latent heat flux (*λ* is the latent heat of vaporization and *E* is the actual ET), *R*_*n*_ is the net radiation, *G*_*0*_ is the soil heat flux, and *H* is the sensible heat flux. Calculation methods of above variables were detailed by Su et al. [50].

Comparing with other energy residual methods, the SEBS model improved the following two aspects. First, based on *κB* ^*-1*^ models of full canopy and bare soil surface, it produced reliable estimates of *κB* ^*-1*^ that were partly covered with vegetation, which was used for the scalar roughness height of heat transfer and *H* estimation. Second, every pixel was placed under the dry and wet limits to avoid uncertainty of spatial interpolation of meteorological data. SEBS assumed that the latent heat flux (*λE*) was zero at the dry limit because of the limitation of soil moisture, but it took place at potential rate in the wet limit. As a result, sensible heat flux reached its maximum and minimum values, respectively, which can be expressed as:

$$H_{dry}=R_{n}-G_{0}$$ (2)

$$H_{wet}=R_{n}-G_{0}-\lambda E_{wet}$$ (3)

According to Eqs 1, 2 and 3, the relative evaporative fraction (*EF*_*r*_) and evaporative fraction (*EF*) then can be calculated as:

$$EF_{r}=\frac{\lambda E}{\lambda E_{wet}}=1-\frac{H-H_{wet}}{H_{dry}-H_{wet}}$$ (4)

$$EF=\frac{\lambda E}{R_{n}-G_{0}}=\frac{EF_{r}\lambda E_{wet}}{R_{n}-G_{0}}$$ (5)

By inverting Eq 5, the actual latent heat flux can be obtained, then daily ET can be calculated as:

$$ET_{daily}=8.64\times 10^{7}\times EF_{daily}\times \frac{R_{n}-G_{0}}{\lambda \rho_{w}}$$ (6)

where *ET*_*daily*_ is the actual daily evapotranspiration (mm/d), *ρ*_*w*_ is the water density. Here, *R*_*n*_ represents daily mean net radiation, and G_0_ is daily mean soil heat flux which is close to zero. *EF*_*daily*_ is the daily mean evaporation fraction. *EF*_*daily*_ can be replaced by *EF* as the model assumes that *EF* remained relatively stable during one day.

We multiplied the reference ET fraction by ET_0_ and completed the extension at the appropriate time scale. The Food and Agriculture Organization (FAO) of the United Nations' report FAO-56 defined ET_0_ as the ET rate from a reference surface that does not experience water shortages [51], and it is calculated as follows:

$$ET_{0}=\frac{0.408\Delta \left(R_{n}-G_{0}\right)+\gamma \frac{900}{T+273}v_{2}\left(e_{s}-e_{a}\right)}{\Delta +\gamma \left(1+0.34v_{2}\right)}$$ (7)

This extension method originated with the model mapping ET with internalized calibration (METRIC) [52], which is similar to the K_C_-ET_0_ approach [51]. Considering the minimal variation in reference ET fraction over several weeks, we can use the following equation to determine the total monthly or seasonal ET:

$$ET_{period}=\sum_{i=m}^{n}\left[\left(ET_{r}F_{i}\right)\left(ET_{0i}\right)\right]$$ (8)

where *ET*_*period*_ is the cumulative ET, *ET*_*r*_ *F* is the reference ET fraction resulting from ET calculated by SEBS over the reference ET (*ET*_*r*_), *ET*_*r*_ *F*_*i*_ is the interpolated *ET*_*r*_ *F* for day *i* and *ET*_*0i*_ is the daily ET_0_ for day *i*.

The precipitation value was obtained from the meteorological station in this area and interpolated to polygon data at the regional scale with the inverse distance weighted method. WY was calculated by determining the precipitation and subtracting the ET.

### Estimate precision of the SEBS model

Because of the lack of availability of ET field data, free water surface evaporation data measured by evaporators at meteorological stations were used to validate the precision of the estimated ET. A 20 m^2^ evaporation tank was recommend by the World Meteorological Organization as the standard evaporimeter to measure free water surface evaporation [53]. In China modified GGI-3000, named E601 evaporator (area 0.3 m^2^), is a common tool used to observe evaporation in meteorological stations [54]. We used the water surface evaporation of Erhai Lake obtained from the adjacent Dali meteorological station in NW Yunnan to estimate the precision of the SEBS model. The converted coefficient between the 20 m^2^ evaporation tank and E601 was 0.97 in Dali [55]. The root mean square deviation (RMSD) and mean absolute percent error (MAPE) were used to compare the differences between the estimated ET and measured value that was converted to the ET of the 20 m^2^ evaporation tank according to the coefficient. These two variables were calculated as follows:

$$RMSD={\left|\frac{1}{n}\sum_{i=1}^{n}{\left(P_{i}-O_{i}\right)}^{2}\right|}^{1/2}$$ (9)

$$MAPE=\frac{100}{n}\sum_{i=1}^{n}\frac{\left|P_{i}-O_{i}\right|}{O_{i}}$$ (10)

where *n* is the number of samples, *P*_*i*_ is the evaporation estimated by the SEBS model, and *O*_*i*_ is the observed value by the E601 evaporator.

### Image processing

All of the obtained images were reprojected and converted to geographic longitude/latitude WGS-1984 projections in geo-tiff format using the MODIS Swath tool. MOD021KM and MOD03 products were used to calculate the surface physical parameters, such as albedo, vegetation coverage, emissivity, surface temperature, and instantaneous normalized difference vegetation index (NDVI), via ILWIS 3.8.3 and ArcGIS 10.

Monthly NDVI data were used to estimate the vegetation coverage based on the dimidiate pixel model. In the dimidiate pixel model, a pixel consists of vegetation and non-vegetation. Thus, the ratio of vegetation coverage to the pixel (vegetation fraction) represents the vegetation coverage, and this model is widely used to estimate vegetation coverage [56, 57]. This variable was calculated as follows [58, 59]:

$$F_{c}=\left(NDVI-NDVI_{soil}\right)/\left(NDVI_{veg}-NDVI_{soil}\right)$$ (11)

where *NDVI*_*soil*_ is the NDVI value of bare soil or non-vegetation coverage, and *NDVI*_*veg*_ represents the NDVI value of full vegetation coverage at a pixel. Given 5% vote of confidence, *NDVI*_*veg*_ and *NDVI*_*soil*_ were set as the maximal and minimal NDVI, respectively [59].

All of the meteorological data were interpolated into gridded data at a spatial resolution of 0.01 degree cell size with the inverse distance weighted method. These data were used in the SEBS model to calculate the relative evaporation and daily ET as well as in the calculations for the reference ET (ET_0_) in the ET_0_ calculator.

### Statistical analysis

Linear trend estimation was employed to quantify the ET and WY trends in every pixel as follows:

$$K=\frac{n\times \sum_{i=1}^{n}i\times S_{i}-(\sum_{i=1}^{n}i)(\sum_{i=1}^{n}S_{i})}{n\times \sum_{i=1}^{n}i^{2}-{\left(\sum_{i=1}^{n}i\right)}^{2}}$$ (12)

where *S*_*i*_ is the cumulative ET or WY and *i* is the sequence number of years (i.e., 1 to *n*). *K > 0* and *K < 0* indicate that the ET or WY of this pixel increases or decreases over time, respectively.

A multivariate linear regression analysis was used to measure the contribution of climatic factors and vegetation changes to ET, and vegetation coverage was considered the fixed variable of the stepwise regression equation. Moreover, the main climatic factors were filtered by the stepwise backwards selection method based on the analysis principle of significance, which could eliminate climate variables with minimal predictive effect. Then, the absolute value of the standardization regression coefficient was used to compare the degree to which vegetation and climate affected ET. Thus, the ratio of the absolute values of specific variables to the sum of the absolute values of all variables was the contribution proportion of this variable. Excel 2010 and SPSS 22 were used for the statistical analyses. All of the figures were drawn in Origin 9.2.

## Results

### Results of model validation

**Fig 2:**
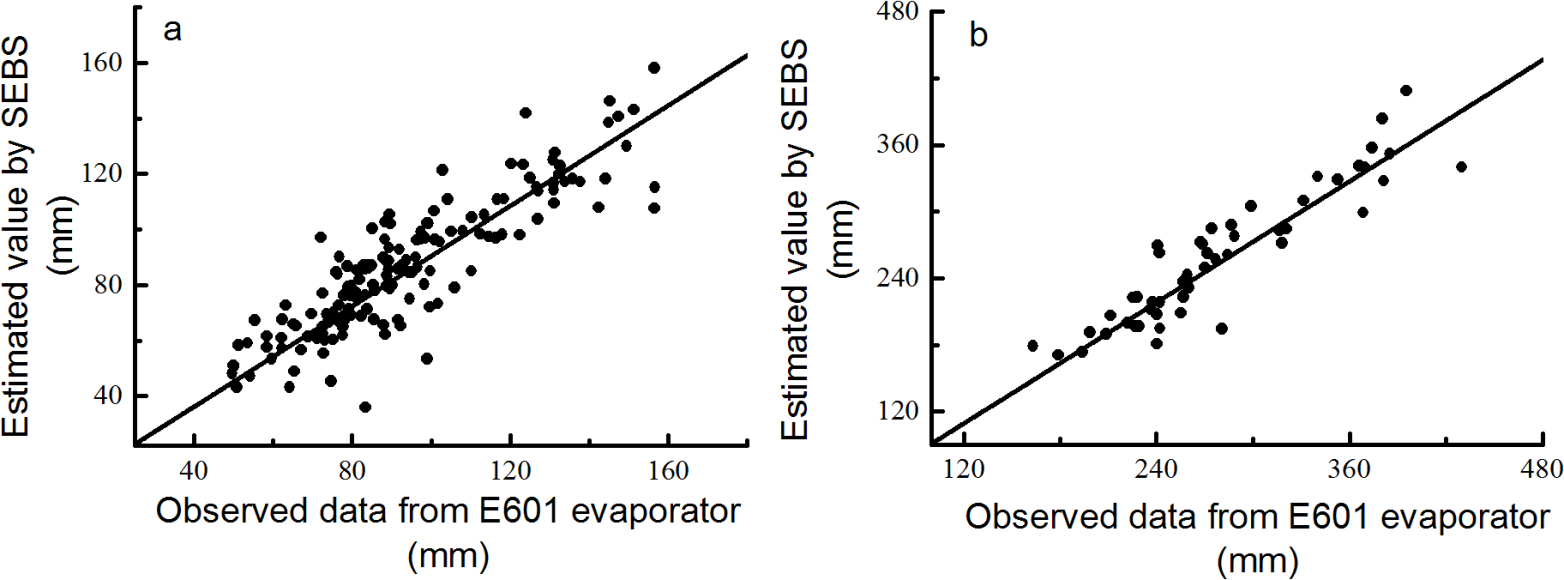
Comparison of water surface evaporation between the SEBS model estimation and observed data from E601. Comparison of water surface evaporation over Erhai Lake between the estimated values and actual observed values at different time scales: (a) monthly water surface evaporation, (b) seasonal water surface evaporation.

Fig 2 shows that the estimated ET was consistent with the value observed by the E601 evaporator. Monthly evaporation had an RMSD of 14.24 mm and a MAPE of 11.28%, whereas seasonal evaporation had an RMSD of 32.05 mm and a MAPE of 9.08%. The correlation coefficient between the model-estimated and observed data reached 0.876 and 0.921 at monthly and seasonal time scales, respectively. Therefore, we concluded that the estimated ET by the SEBS model matched the actual situation in NW Yunnan.

### Interannual changes in climatic factors and vegetation coverage

**Fig 3:**
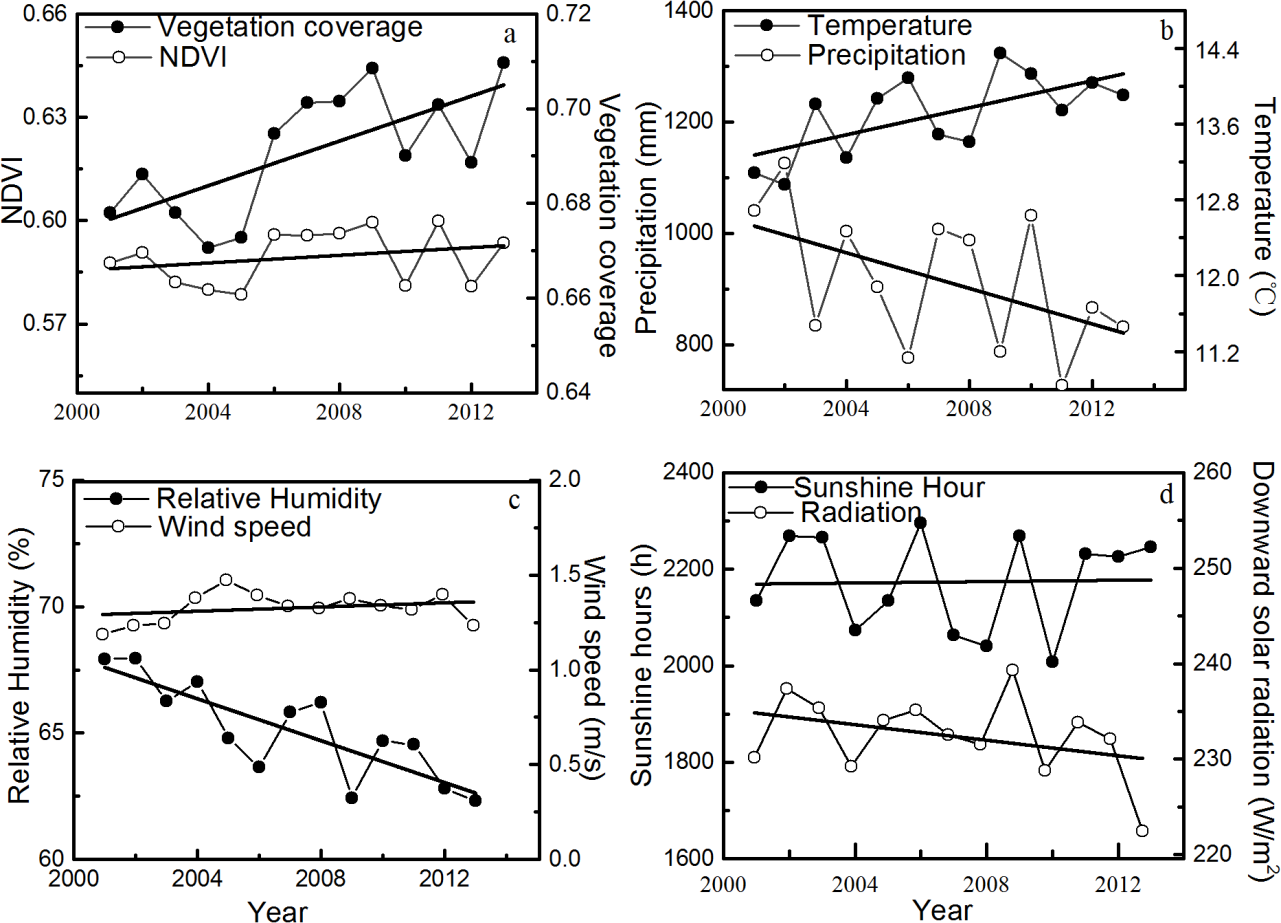
Interannual variations of climate and vegetation coverage. Interannual variations of climate and vegetation coverage: (a) Vegetation coverage and NDVI, (b) temperature and precipitation, (c) relative humidity and wind speed, and (d) sunshine hours and downward solar radiation. Each part of the figure has double Y axes. Each black line represents the linear fit of each variable as a function of the year. Significance tests show that the P-values of (a) vegetation coverage, (b) temperature, and (c) relative humidity were 0.008, 0.017, and 0.000, respectively, whereas the P-values of all other variables were greater than 0.05.

The average annual precipitation and temperature of NW Yunnan varied from 789.41 mm to 1,125.26 mm and from 12.95°C to 14.34°C over the last 13 years, respectively. The linear trend estimation indicated that the temperature significantly increased over time (P < 0.05) and the precipitation fluctuated downward but was not statistically significant (Fig 3B). In addition, the average annual relative humidity significantly decreased (R^2^ = 0.683, P < 0.01). Other climatic factors (average wind speed at reference height, total sunshine hours, and downward solar radiation) fluctuated over time without clear trends (Fig 3C and 3D). A correlation analysis revealed that the precipitation was positively correlated with relative humidity and negatively correlated with temperature, sunshine hours, and air pressure, whereas air temperature was negatively correlated with relative humidity at the 0.01 significance level (Table 1). The average annual vegetation coverage was 0.69, ranged from 0.67 to 0.72, and showed a significant increasing trend (P < 0.01) over time. However, the NDVI fluctuated without an overall trend.

**Table 1:** Pearson correlation coefficient matrix of climate variables.

	Precipitation	T_air_ ^a^	RH^b^	V_wind_ ^c^	P_air_ ^d^	H_sunshine_ ^e^	Ra^f^
**Precipitation**	1						
**T**_**air**_	-0.713**	1					
**RH**	0.736**	-0.890**	1				
**V**_**wind**_	-0.291	0.508	-0.434	1			
**P**_**air**_	-0.559*	0.660*	-0.574*	0.603*	1		
**H**_**sunshine**_	-0.590*	0.261	-0.361	-0.16	-0.144	1	
**Ra**	-0.15	0.086	0.102	0.248	-0.136	0.405	1

^a^Mean annual air temperature

^b^relative humidity

^c^wind speed at the reference height

^d^air pressure

^e^total annual sunshine hours, and

^f^downward solar radiation.

Asterisks indicate significant differences for climate variables

* indicates *P* < 0.05

** indicates *P* < 0.01.

### Interannual changes in ET and WY

**Fig 4:**
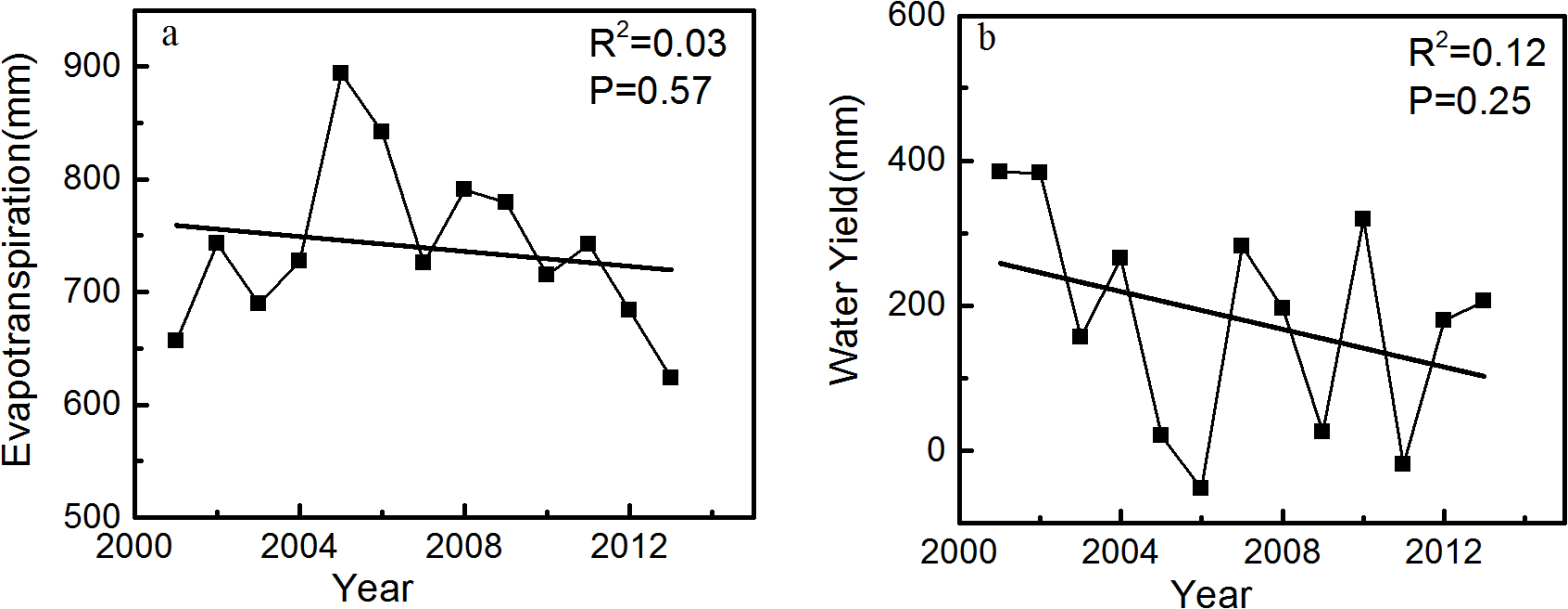
Interannual variations of ET and WY. Interannual variations of ET and WY: (a) ET and (b) WY.

The average annual ET (739.27 mm) was lower than the precipitation (919.74 mm) in this area during the study period. However, the ET reached 841.30 and 742.35 mm and the precipitation was only 789.41 and 723.41 mm in 2006 and 2011, respectively, which decreased the WY to below 0 (Fig 4B). The mean ET rate (ratio of ET to precipitation (ET/P)) was 0.80, whereas the WY rate (ratio of WY to precipitation (WY/P)) was only 0.20. Both ET and WY presented considerable fluctuations, with ranging from 623.29 mm to 893.8 mm and −51.88 mm to 384.40 mm, respectively. The ET and WY rates ranged from 0.63 to 1.06 and −0.06 to 0.36, respectively.

### Seasonal changes in ET and WY

**Fig 5:**
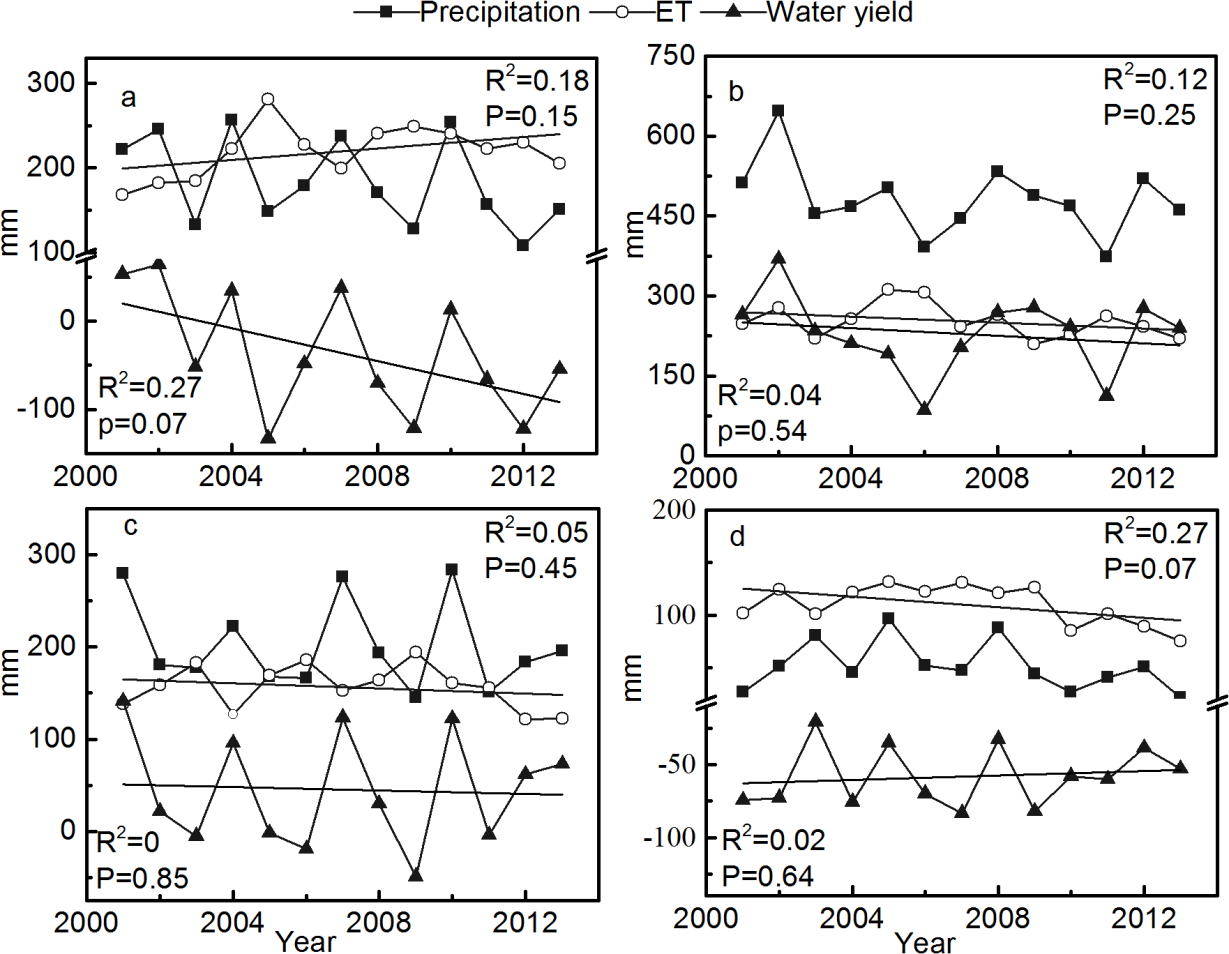
Seasonal changes of ET and WY. Seasonal changes of ET and WY: (a) spring, (b) summer, (c) autumn, and (d) winter. The unit of ET, WY, and precipitation is mm per three months. The trends of seasonal ET and WY are analyzed by linear trend estimation.

In general, the highest and lowest ET, precipitation, and WY were recorded in summer and winter, respectively. The mean ET values in spring, summer, autumn, and winter were 219.60, 252.95, 156.50, and 110.21 mm, respectively. Furthermore, the mean ET values in spring and winter were higher than the precipitation values, resulting in higher ET rates and lower WY rates. The average ET rates in spring, summer, autumn, and winter were 1.30, 0.53, 0.83, and 2.42, respectively, and significant trends were not observed. The WYs of the four seasons also did not show significant trends during the research period, although the WYs in spring decreased more obviously than in any other season because of increasing ET and decreasing precipitation (Fig 5).

### Spatial variation of ET and WY

**Fig 6:**
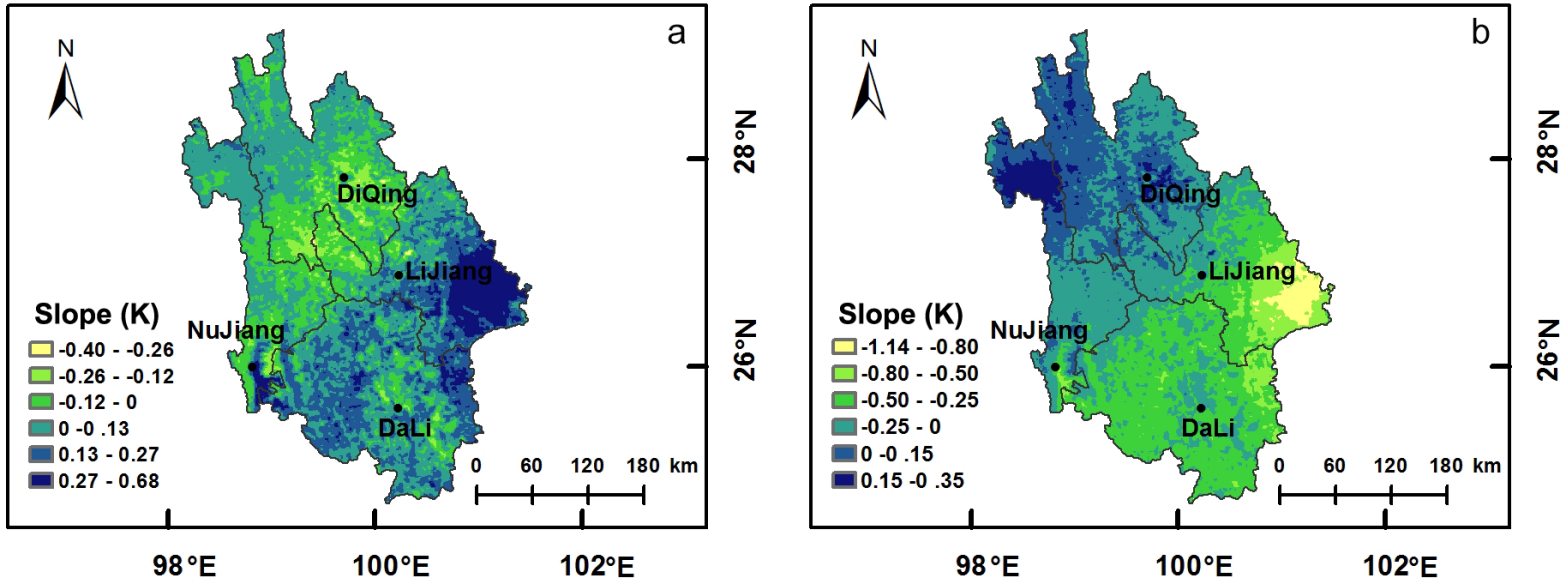
Spatial variations of ET and WY. Maps displaying the slope of ET and WY by linear trend estimation: (a) ET and (b) WY. K > 0 and K < 0 in any pixels indicate increasing and decreasing trends over time, respectively.

Fig 6 illustrates the variations in ET and WY with time in NW Yunnan. The results of the linear least squares method show that the ET slope exceeded 0 in the southeast area of NW Yunnan, which was primarily in the Dali Bai Autonomous Prefecture and Lijiang City. However, this slope was less than 0 in northwest area of NW Yunnan, which was inaccessible and geographically remote (Fig 6A). Thus, the ET in Dali and Lijiang increased with time, whereas it decreased with time in Diqing and Nujiang. Moreover, F-test showed that ET significantly increased in Lijiang (S1a Fig), where the vegetation coverage also had a significant increase trend (Fig 1). On the other hand, the trend of WY was almost the exact opposite to that of ET (Fig 6B and S1b Fig), which suggests that WY decreased with increasing ET.

### Correlations of ET with climate factors and vegetation coverages

Annual time series data of ET and WY were used for multivariate linear regression analysis. The standardized regression equations are listed in Table 2, and the R^2^ values of the regression equations were 0.76, 0.88, 0.90, and 0.88, and each equation reached a significant level (Table 2). The variance inflation factor (VIF) of all variables in the four regression equations was less than 5, which indicated minimal multicollinearity among independent variables. The absolute values of the independent variables in the ET regression equation were ordered as follows: wind speed > relative humidity > vegetation coverage > sunshine hours. Vegetation coverage accounted for 14.82% of the ET (0.426/2.874), and all climatic factors accounted for 85.18% (2.448/2.874) of the ET, which indicated that climatic factors (wind speed, relative humidity, and sunshine hours) were the main cause of ET and vegetation coverage played an integral, although not decisive, role. ET rates, WY, and WY rates were mainly affected by climatic factors, which accounted for 98.92%, 98.88%, and 98.92% of the values, respectively. Precipitation was the main factor that influenced ET rates, WY, and WY rates, and the ratios of precipitation to the total regression coefficient were 53.1%, 67.5%, and 53.1%, respectively. Moreover, precipitation was positively correlated with the WY and WY rate but negatively correlated with the ET rate. This result indicated that the ET rate increased and WY decreased with decreasing precipitation.

**Table 2:** Regression analysis of ET and WY.

Dependent variable	Standardized regression equation	R^2^	F	P
**ET**	Y = 0.856RH^a^+1.199V_wind_ ^b^+0.393H_sunshine_ ^c^+0.426F_c_ ^d^	0.758	6.272	0.014
**ET rate**	Y = -0.686P^e^+0.331V_wind_+0.260Ra^f^+0.014F_c_	0.883	15.09	0.001
**WY**	Y = 0.782P-0.362V_wind_+0.013F_c_	0.901	27.169	0.000
**WY rate**	Y = 0.686P-0.331V_wind_-0.260Ra-0.014F_c_	0.883	15.09	0.001

^a^Relative humidity

^b^wind speed at the reference height

^c^total annual sunshine hours

^d^vegetation fraction

^e^precipitation, and

^f^downward solar radiation.

## Discussion

Studies have shown that anomalous monsoons induced by ENSO cause weak atmospheric circulation, which results in precipitation deficits in Yunnan [16, 19, 23]. Li et al. (2011) also found that precipitation decreased in the Hengduan Mountains after 2000 [60], which was likely caused by the warm and dry climate [17]. From the perspective of WY, We found that climatic factors (precipitation) greatly influenced the WY (Table 2), and the precipitation deficiency could be considered a main reason for the droughts, which is consistent with the findings of Lü et al. (2012), who studied the physical causes of the severe droughts in the region and drew the same conclusion [18].

In addition, vegetation coverage also explained the changes in WY or ET (Table 2). We found that the contribution of vegetation coverage was small (14.82%) but could not be ignored. Theoretically, climatic factors and human activities are both responsible for vegetation coverage changes, e.g., temperature affects plant phenology or vegetation seasonal dynamics [61, 62], and precipitation positively correlates with vegetation coverage [63, 64]. In NW Yunnan, vegetation coverage markedly increased when precipitation decreased (Table 1), indicating that precipitation was not the major cause of the increased vegetation coverage. We believe that the afforestation/reforestation in recent decades has been the driver of vegetation recovery in Yunnan which has experienced an increase in forest cover from 34% in 1997 to 50% in 2006 with afforestation programs, such as the Natural Forest Protection Program [65]. Plantation forests cover 3.26 million ha, which only account for 17.97% of the total woodland, but the annual growth rate is 17.72% which is greater than that of natural forests (4.08%) [66]. Moreover, Greenpeace indicated that most of the primary forests were replaced by secondary forests (71.18%) and artificial forest (approximately 20%) [32]. Thus, the forest quality deteriorated, despite the cover increasing in recent decades [31, 67]. Studies have shown that the increased ET caused by afforestation/reforestation could reduce WY or runoff [26, 68, 69] and the ET of fast-growing plantation forests was much higher than that of natural primary forests [70–73]. Moreover, in NW Yunnan, remarkable encroachment of woody-plants has been observed in alpine meadows from 1950 to 2009, and at least 39% of the alpine meadows have been converted to woody shrubs [74]. The encroachment of woody plants could also increase the ET of an ecosystem [69].

Spatio-temporal variability of ET and WY matched the actual drought events in Dali and Lijiang in springs (CCTV: http://english.cntv.cn/program/china24/20120214/110226.shtml). Dali and Lijiang had a large area of secondary forests of Yunnan pine (*Pinus yunnanensis*), a tree species that is commonly used in afforestation/reforestation [36, 75], whereas the primary natural forests mainly occurred in remote areas, such as Nujiang and Diqing. Differences in the location of primary and secondary growth forests might be one of the reasons for differentiated ET among these prefectures. Although the transpiration data for Yunnan pine forests were unavailable, pine artificial forests have been proven to have higher water consumption than natural forests worldwide [70, 71]. However, Zhang et al. (2011) found that primary and old-growth dark coniferous forests in Southwest China had a lower annual ET and a higher annual WY than shrub lands, regenerated forests, and spruce plantations [76]. Therefore, attention should be paid to ecohydrological effects of human-driven land-cover changes, because higher ET usually implies less water available for human use [77].

Local human populations rely heavily on and are constrained by water resources. Government statistics [40, 78] and water use quota [79] were used in calculations according to Qin et al. (2014) [80], and we found that the water consumption for primary industry, second industry, tertiary industry and domestic use already accounted for 49.15% of the WY. Primary industry and domestic uses accounted for 75.94% of the total demands and only 9.43% of the total demands was used for industry development. These results indicate that most of the WY was used for subsistence and agricultural production. Because of the poor infrastructure used to extract river water from upstream areas, water provisions from local ecosystems are essential in the mountainous areas of NW Yunnan. Therefore, even a slight ET increase as a result of vegetation changes could significantly increase the system's vulnerability to climate change, especially in rural areas.

## Conclusion

Water deficiencies caused by abnormal climate were the primary cause of continuous droughts in NW Yunnan; however, the increased ET resulting from human-driven land-cover and vegetation changes also exacerbated the water shortages. Local human systems are highly vulnerable to climatic drought in term of the water resources provided by its ecosystems. Human disturbances to natural forests as well as massive afforestation/reforestation with several fast-growing tree species are harmful to native biodiversity and increase the system’s vulnerability to climate change. In addition to developing infrastructure to mitigate the impacts of droughts, such as irrigation systems and reservoirs, we strongly suggest increasing the conservation efforts related to native vegetation and developing water-conserving agricultural techniques as adaptive strategies to climate change.

## Supporting Information

S1 Fig The trend of ET and WY from 2001 to 2013. The trend of ET and WY: (a) the trend of ET, (b) the trend of WY. F-test is used to test significance of the slopes in Fig 6. The maps are reclassified into different categories according to different significance levels: very significantly (P < 0.01), significantly (P < 0.05), and slightly (P > 0.05).
(TIF)
