# Supplementary figures and images for: Ecosystem Evapotranspiration as a Response to Climate and Vegetation Coverage Changes in Northwest Yunnan, China
Source: PLoS One. 2015 Aug 3;10(8):e0134795. doi: 10.1371/journal.pone.0134795 (PMC4523184; doi:10.1371/journal.pone.0134795)

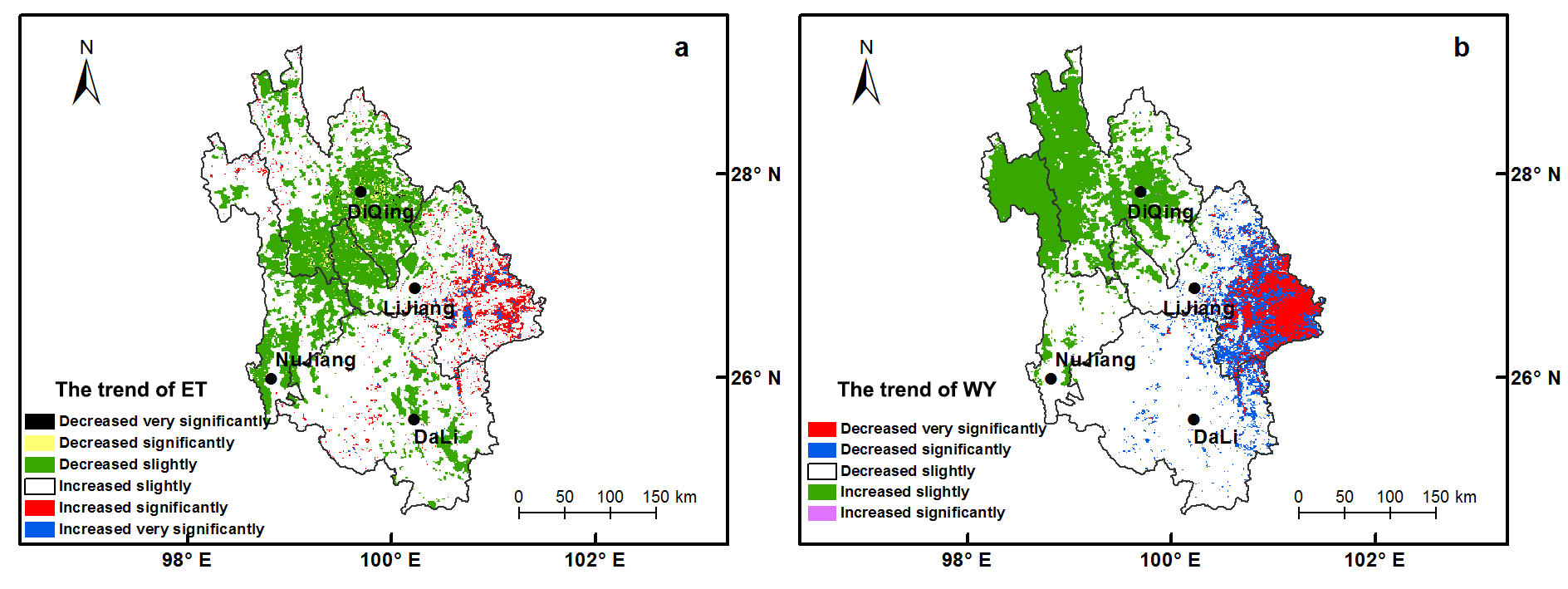

Supplement: S1 Fig — The trend of ET and WY: (a) the trend of ET, (b) the trend of WY. F-test is used to test significance of the slopes in Fig 6. The maps are reclassified into different categories according to different significance levels: very significantly (P < 0.01), significantly (P < 0.05), and slightly (P > 0.05). (TIF) [file pone.0134795.s001.tif]
